# Clinical and prognostic factors associated with diagnostic wait times by breast cancer detection method

**DOI:** 10.1186/2193-1801-3-125

**Published:** 2014-03-06

**Authors:** Amalia Plotogea, Anna M Chiarelli, Lucia Mirea, Maegan V Prummel, Nelson Chong, Rene S Shumak, Frances P O’Malley, Claire MB Holloway, The Breast Screening Study Group

**Affiliations:** Prevention and Cancer Control, Cancer Care Ontario, Toronto, Canada; Dalla Lana School of Public Health, University of Toronto, Toronto, Canada; Maternal-Infant Care Research Centre, Mount Sinai Hospital, Toronto, Canada; Institute for Clinical Evaluative Sciences, Toronto, Canada; Department of Laboratory Medicine and Pathobiology, University of Toronto and St. Michaels Hospital, Toronto, Canada; Women’s College Hospital Sunnybrook Health Sciences Centre, Toronto, Canada

**Keywords:** Breast cancer, Diagnostic wait time, Mammography, Interval cancers, Symptomatic cancers, Screen-detected cancers

## Abstract

**Introduction:**

Although prognostic differences between screen-detected, interval and symptomatic breast cancers are known, factors associated with wait times to diagnosis among these three groups have not been studied.

**Methods:**

Of the 16,373 invasive breast cancers diagnosed between January 1, 1995 and December 31, 2003 in a cohort of Ontario women aged 50 to 69, a random sample (N = 2,615) were selected for chart abstraction. Eligible women were classified according to detection method; screen-detected (n = 1181), interval (n = 319) or symptomatic (n = 406). Diagnostic wait time was calculated from the initial imaging or biopsy to breast cancer diagnosis. Logistic regression analysis examined associations between diagnostic wait times dichotomized as greater or less than the median and demographic, clinical and prognostic factors separately for each detection cohort.

**Results:**

Women who underwent an open biopsy had significantly longer than median wait times to diagnosis, compared to women who underwent a fine needle aspiration or core biopsy; (screen-detected OR = 2.76, 95% CI = 2.14-3.56; interval OR = 2.56, 95% CI = 1.50-4.35; symptomatic OR = 5.56, 95% CI = 3.33-9.30). Additionally, screen-detected breast cancers diagnosed with stage II and symptomatic cancers diagnosed at stage III or IV had significantly shorter diagnostic wait times compared to those diagnosed at stage 1 (OR = 0.66 95% CI = 0.50-0.87 and OR = 0.46, 95% CI = 0.25-0.85 respectively).

**Conclusions:**

Our study is consistent with expedited diagnostic work-up for breast cancers with more advanced prognostic features. Furthermore, women who had an open surgical biopsy had a greater than the median diagnostic wait time, irrespective of detection method.

## Introduction

Delays in assessment of an abnormal mammogram have been shown to be associated with patient stress and anxiety (Rimer and Bluman 1997; Brett et al. 1998; Sutton et al. 1995). A prolonged assessment pathway may also impact prognosis in women where the final diagnosis is cancer (Ganry et al. 2004; Olivotto et al. 2002). Data from Canadian screening programs suggest that delays in breast cancer diagnosis beyond 20 weeks after an abnormal screen are associated with an increased likelihood of lymph node metastases and increased tumour size compared with breast cancers diagnosed between 4 and 12 weeks (Olivotto et al. 2002). Another study from France also reported that longer intervals to screen-detected breast cancer were associated with increasing risk of lymph node metastases and larger tumour size (Ganry et al. 2004). A systematic review of symptomatic breast cancers noted that delays from symptoms to treatment of 3 to 6 months resulted in significantly lower survival rates for breast cancer patients (Richards et al. 1999).

For women with screen-detected breast cancer, longer intervals to diagnosis have been associated with living in urban versus rural areas (Caplan et al. 2000). Several studies have also found that among women with screen-detected breast cancer, those with “high-suspicion” compared to “low suspicion” screening mammograms had shorter diagnostic intervals (Olivotto et al. 2002; Ganry et al. 2004; Caplan et al. 2000). A study of women with abnormalities detected through Canadian screening programs reported longer median waiting times for a diagnosis when an open biopsy was performed; however wait times were shorter for programs that used core biopsies more often (vOlivotto et al. 2001).

Numerous studies have examined prognostic differences between screen-detected, interval and symptomatic breast cancers (Burrell et al. 1996; Schroen et al. 1996; Dillon et al. 2004; Burke et al. 2008; Chiarelli et al. 2012). In our previously conducted study, screen-detected cancers were found to have more favourable prognostic features compared to symptomatic or interval cancers, while interval cancers had intermediate prognostic features compared to tumours detected by screening or without screening (Chiarelli et al. 2012). However, no studies have compared factors associated with diagnostic wait times by detection method. The purpose of this study is to identify demographic, clinical and prognostic factors associated with median diagnostic wait times separately for each cohort (screen-detected, interval and symptomatic) by detection method.

## Methods

### Study population

Ethics approval for this study was granted from the University of Toronto Health Sciences Research Board, the Regional Cancer Centers (RCC) and Princess Margaret Hospital (PMH). Methods were described thoroughly in Chiarelli et al. (2012). Briefly, a cohort of women (N = 807,966) between the ages of 50–63 as of Jan 1, 1995 who registered for health care benefits through the Ontario Health Insurance Plan (OHIP) was identified. The cohort was linked to women in the Ontario Cancer Registry (OCR) to ascertain invasive primary breast cancer, of any histological type, from Jan 1, 1995 to Dec 31, 2003. Women identified with prior history of breast cancer (n = 15,684), unknown sex (n = 3), who were not residents of Ontario (n = 7,633), less than 50 years of age (n = 33) or who had died before the start of the study (n = 12,898) were excluded.

Information on mammograms performed through OHIP was obtained by merging cohort data with OHIP files and extracting all physician claims for bilateral mammography during study period with an algorithm to distinguish between screening and diagnostic mammograms (Chiarelli et al. 2012). Information from women screened within the Ontario Breast Screening Program (OBSP) was obtained from data routinely collected by an integrated client management system. Since 1990, the OBSP has offered eligible women biennial screening consisting of two view mammography. A complete description of the details of the OBSP has been published (Chiarelli et al. 2006). All identified OBSP and OHIP screens were merged to obtain a complete screening history for each woman.

**Figure 1:**
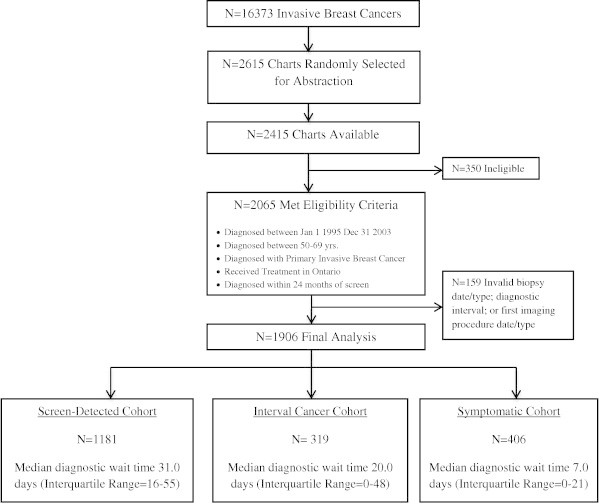
**Flow chart of study population leading to final cohort with median delay for the 3 detection cohorts.**

Of the 16,373 invasive breast cancer cases that occurred in Ontario between 1995 and 2003, 2,615 were randomly selected for chart abstraction. Of the 2,415 women with available charts, 350 did not meet the eligibility criteria (specified in Figure 1). A woman’s breast cancer was classified as screen-detected if she had a mammogram during the study period, either through OHIP or OBSP, and her breast cancer was diagnosed within 6 months of that screen. Interval breast cancers were defined as cancers that occurred within 6 to12 months of a mammogram. Symptomatic breast cancers were cancers detected in women who did not have a screening mammogram during the study period prior to diagnosis.

### Definition of diagnostic wait times

Mammography screening dates were obtained from the OBSP or OHIP administrative databases and information on assessment dates and procedures was abstracted from imaging, biopsy and surgical reports within medical charts (Chiarelli et al. 2012). For women with screen-detected cancers, the diagnostic wait time commenced with the date of the last screening mammogram preceding diagnosis. For interval cancers, the diagnostic wait time commenced with the date of the first imaging procedure (n = 275, 86.2%) or biopsy (n = 98, 24.1%), whichever came first, following the screening mammogram. Similarly, for symptomatic cancers, it began with the date of the first imaging procedure (n = 308, 75.9%) or biopsy (n = 44, 13.8%), whichever came first (Gorin et al. 2006). Breast imaging included mammogram, chest ultrasound, chest x-ray or imaging consultation. A breast biopsy included fine needle aspiration (FNA) or core biopsy. The date of definitive breast cancer diagnosis was used as the end of the diagnostic interval for all women.

### Definition of covariates

Information on tumour characteristics was abstracted from pathology and surgical reports included in the medical charts (Chiarelli et al. 2012). The TNM classification scheme was used for staging of breast cancer (American Joint Committee on Cancer 2002). Tumour size was defined as the largest diameter of the invasive carcinoma. Among women who had axillary assessment with either sentinel lymph node biopsy or axillary node dissection, lymph node status was defined as positive by TNM criteria. OCR data was used to obtain age and date at diagnosis and treatment center location. Treatment center was classified as the cancer center the woman first attended, and grouped by region in Ontario. South Central region included Toronto and Hamilton, South Eastern region included Ottawa and Kingston areas, South Western region included London and Windsor areas, and Northern region included Thunder Bay and Sudbury areas. Population level factors, including neighbourhood income quintile, and community size were obtained from the 2001 Canadian Census. Postal codes from either residence at first screen (interval and screen-detected cases) or the start of the study period, January 1, 1995 (symptomatic cases) were linked to the Canadian census, to obtain average household income figures and census population information (Chiarelli et al. 2012). Biopsies included those conducted within a day of the diagnosis date and were either percutaneous, (FNA and core biopsies) or surgical (excisional biopsy or partial mastectomy).

### Statistical analysis

Association of demographic, clinical and prognostic characteristics with detection method was examined using polytomous logistic regression comparing interval and symptomatic cancers to screen-detected cancers. Logistic regression analysis examined associations between factors and diagnostic wait times, dichotomized as greater or less than the observed median, separately for each detection group. Adjusted odds ratios (OR) and 95% confidence intervals (95% CI) were estimated to quantify associations. All statistical analyses were performed by SAS 9.2 (SAS Institute) and statistical significance was evaluated using 2-sided p-values at the 5% testing level (SAS Institute Inc 2008).

## Results

The final sample was comprised of 1,181 (97.4%) screen-detected, 319 (88.1%) interval and 406 (82.7%) symptomatic breast cancers (Figure 1). Median diagnostic wait times were 31 days for screen-detected cancers (Interquartile Range (IQR) =16-55), 20 days for interval cancers (IQR = 0-48) and 7 days for symptomatic cancers (IQR = 0-21).

Compared to screen-detected cancers, women with symptomatic detected cancer were less likely to live in a high income neighbourhood (highest vs. lowest OR = 0.59, 95% CI =0.42-0.84), or undergo an open biopsy (OR = 0.56, 95% CI =0.44-0.72), and more likely to be diagnosed with a larger tumour size (> 2.0 cm vs. <1.0 cm OR = 6.92 95% CI =4.74-10.10), with positive nodes (OR = 1.96, 95% CI =1.52-2.52), and at a higher stage (III + IV vs. I OR = 6.82, 95% CI =4.89-9.50) (Table 1). Interval cancers were also more likely than screen-detected cancers to be diagnosed at larger tumour size (OR = 1.68, 95% CI =1.21-2.35) and at a higher stage (OR = 1.99, 95% CI =1.40-2.84).

**Table 1:** **Adjusted odds ratio (OR) and 95% confidence intervals (CIs) of demographic and tumour characteristics among interval, and symptomatic detected breast cancers compared to screen-detected cancers**

	Screen-detected (N = 1181)	Interval (N = 319)	OR^a^(95% CI)	Symptomatic (N = 406)	OR^a^(95% CI)
Characteristics	n (%)	n (%)		n (%)	
**Age at diagnosis** ^**a**^					
50-59	450(38.1)	122(38.2)	1.00(reference)	165(40.6)	1.00(reference)
60-69	731(61.9)	197(61.8)	0.90 (0.58-1.40)	241(59.4)	1.11(0.74-1.68)
**Last screen** ^**a**^					
OBSP	427(36.2)	123(38.6)	1.00(reference)	--	--
OHIP	754(63.8)	196(61.4)	0.98(0.75-1.26)	--	--
**Period of diagnosis** ^**a**^					
1995-1999	652(55.2)	148(46.4)	1.00(reference)	246(60.6)	1.00(reference)
2000-2003	529(44.8)	171(53.6)	0.64 (0.39-1.05)	160(39.4)	0.83 (0.53-1.29)
**Treatment center region** ^**a**^					
South Central	530(44.8)	150(47.0)	1.00(reference)	193(47.5)	1.00 (reference)
South Eastern	297(25.2)	70(21.9)	0.79(0.58-1.09)	95(23.4)	0.90(0.68-1.19)
South Western	219(18.5)	56(17.6)	0.88(0.62-1.25)	71(17.5)	0.91(0.66-1.25)
Northern	135(11.4)	43(13.5)	1.06(0.72-1.57)	47(11.6)	0.99(0.68-1.44)
**Income quintiles** ^**a**^					
1-lowest	185(15.7)	43(13.7)	1.00(reference)	94(23.6)	1.00(reference)
2	221(18.8)	56(17.8)	1.04(0.67-1.62)	71(17.8)	0.62(0.45-0.92)*
3	224(19.0)	72(22.9)	1.34(0.87-2.05)	64(16.0)	0.56(0.39-0.82)*
4	246(20.9)	51(16.2)	0.85(0.54-1.33)	78(19.6)	0.63(0.44-0.89)*
5-highest	301(25.6)	92(29.3)	1.31(0.87-1.97)	92(23.1)	0.59(0.42-0.84)*
*Missing*	*4*	*5*		*7*	
**Community size** ^**a**^					
1 500 000+	358(30.3)	109(34.2)	1.00(reference)	139(34.2)	1.00(reference)
500 000–1 499 999	205(17.4)	45(14.1)	0.68(0.46-1.00)	54(13.3)	0.69(0.48-0.99)*
100 000–499 999	297(25.2)	76(23.8)	0.79(0.57-1.10)	78(19.2)	0.69(0.50-0.95)*
10 000 – 99 999	120(10.2)	42(13.7)	1.15(0.76-1.73)	61(15.0)	1.33 (0.92-1.92)
<10 000	201(17.0)	47(14.7)	0.73(0.46-1.07)	74(18.2)	0.97(0.70-1.36)
**Diagnostic biopsy** ^**b**^					
FNA/Core	618(52.3)	180(56.4)	1.00(reference)	259(63.8)	1.00(reference)
Open	563(47.8)	139(43.5)	0.94 (0.72-1.22)	147(36.2)	0.56(0.44-0.72)**
**Tumour size (cm)** ^**c**^					
Mean size at diagnosis	1.83	2.1		3.26	
<1.0	384(33.2)	84(27.5)	1.00(reference)	39(10.6)	1.00(reference)
1.0-1.5	268(23.2)	61(20.0)	1.09(0.75-1.58)	46(12.5)	1.63(1.03-2.57)*
1.5-2.0	209(18.1)	50(16.4)	1.13(0.76-1.68)	62(16.8)	2.61(1.67-4.06)**
>2.0	296(25.6)	110(36.1)	1.68(1.21-2.35)*	222(60.2)	6.92(4.74-10.10)**
*Missing*	*24*	*14*		*37*	
**Node status** ^**c**^					
Negative	779(70.1)	191(66.1)	1.00(reference)	182(52.6)	1.00(reference)
Positive	333(29.9)	98(33.9)	1.20(0.91-1.58)	164(47.4)	1.96(1.52-2.52)**
*Missing*	*69*	*30*		*60*	
**Stage at diagnosis** ^**c**^					
I	662(58.7)	150(49.5)	1.00(reference)	92(24.2)	1.00(reference)
II	332(29.4)	90(29.7)	1.20(0.89-1.62)	159(41.8)	3.19(2.37-4.27)**
III-IV	134(11.9)	63(20.7)	1.99(1.40-2.84)*	129(33.9)	6.82(4.89-9.50)**
*Missing*	*53*	*16*		*26*	

^a^adjusted by age at diagnosis (continuous); year of diagnosis (continuous).

^b^adjusted by age at diagnosis (continuous) year of diagnosis (continuous) treatment center region (categorical) income quintile (categorical).

^c^adjusted by age at diagnosis (continuous) year of diagnosis (continuous) treatment center region (categorical) income quintile (categorical); biopsy type (categorical).

*p < 0.05.

**p < 0.0001.

For screen-detected and interval cancers, women living in smaller communities had a significantly shorter diagnostic wait time, particularly between communities with 500,000-1,499,999 compared to communities >1,500,000 (OR = 0.42, 95% CI = 0.29-0.61, OR = 0.41, 95% CI =0.20-0.85 respectively) (Table 2). For interval cancers, women screened through OHIP had greater diagnostic wait times than those screened through the OBSP (OR = 1.93 95% CI 1.20-3.10). Women with interval and symptomatic detected cancers, who attended a treatment centre in the South Eastern region of the province, had significantly shorter diagnostic wait times (OR = 0.47, 95% CI = 0.25-0.85; OR = 0.58, 95% CI = 0.35-0.97, respectively) and women with symptomatic detected cancers had significantly greater diagnostic wait times if they attended a treatment centre in the Northern region of Ontario (OR = 2.52, 95% CI = 1.26-5.03), compared to those in the South Central region.

**Table 2:** **Adjusted odds ratio (OR) and 95% confidence intervals (CI) of diagnostic wait times by demographic and tumour characteristics for screen, interval and symptomatic detected breast cancers**

	Screen-detected (N = 1181)			Interval (N = 319)			Symptomatic (N = 406)		
Characteristics	n (%)		OR^a^	n (%)		OR^a^	n (%)		OR^a^
Wait time	***<=31 days***	***>31 days***	***(95% CI)***	***<=20 days***	***>20 days***	***(95% CI)***	***<=7 days***	***>7 days***	***(95% CI)***
**Overall**	599(50.7)	582 (49.3)		159 (49.8)	160 (51.2)		207 (49.0)	199 (51.0)	
**Age at diagnosis** ^**a**^									
50-59	232 (38.7)	218 (37.5)	1.00 (reference)	70 (43.8)	52 (32.7)	1.00 (reference)	79 (38.2)	86 (43.2)	1.00 (reference)
60-69	367 (61.3)	364 (62.5)	0.91(0.61-1.37)	90 (56.5)	107 (67.3)	0.55(0.24-1.21)	128 (61.8)	113 (56.8)	1.05(0.50-2.18)
**Period of diagnosis** ^**a**^									
1995-1999	335 (55.9)	317 (54.5)	1.00 (reference)	79(49.4)	69 (43.4)	1.00 (reference)	121 (58.5)	125 (62.8)	1.00 (reference)
2000-2003	264 (44.1)	265 (45.5)	0.73(0.46-1.14)	81(50.6)	90 (56.6)	1.72(0.73-4.07)	86 (41.6)	74 (37.2)	0.58(0.26-1.29)
**Last screen** ^**a**^									
OBSP	206 (34.4)	221 (38.0)	1.00 (reference)	74 (46.3)	49 (30.8)	1.00 (reference)	--	--	--
OHIP	393 (65.6)	361 (62.0)	0.87(0.69-1.13)	86 (53.8)	110 (69.2)	1.93(1.20-3.10)*	--	--	--
**Treatment center region** ^**a**^									
South Central	269 (44.9)	261 (44.9)	1.00 (reference)	71 (44.4)	79 (49.7)	1.00 (reference)	98 (29.5)	95 (47.7)	1.00 (reference)
South Eastern	160 (26.7)	137 (23.5)	0.86(0.65-1.16)	45 (28.1)	25 (15.7)	0.47(0.25-0.85)*	61 (29.5)	34 (17.1)	0.58(0.35-0.97)*
South Western	111 (18.5)	108 (18.6)	0.99(0.72-1.36)	28 (17.5)	28 (17.6)	0.87(0.46-1.63)	34 (16.4)	37 (18.6)	1.17(0.68-2.02)
Northern	59 (9.90)	76 (13.1)	1.29(0.88-1.89)	16 (10.0)	27 (16.9)	1.50(0.73-3.06)	14 (6.8)	33 (16.6)	2.52(1.26-5.03)*
**Income quintiles** ^**a**^									
1	102 (17.1)	83 (14.3)	1.00 (reference)	22 (14.0)	21 (13.4)	1.00 (reference)	51 (25.0)	43 (22.1)	1.00 (reference)
2	102 (17.1)	119 (20.5)	1.43 (0.96-2.11)	25 (15.9)	31 (19.8)	1.25(0.55-2.82)	35 (17.2)	36 (18.5)	1.24 (0.68-2.31)
3	117 (19.6)	107 (18.5)	1.13 (0.76-1.66)	39 (24.8)	33 (21.0)	0.84 (0.39-1.83)	30 (14.7)	34 (17.4)	1.34 (0.71-2.54)
4	115 (19.3)	131 (22.6)	1.40 (0.95-2.05)	27 (17.2)	24 (15.3)	0.86 (0.37-1.98)	44 (21.6)	34 (17.4)	0.89 (0.48-1.63)
5	161 (27.0)	140 (24.1)	1.08 (0.75-1.56)	44 (27.5)	48 (30.6)	1.13 (0.54-2.37)	44 (21.6)	48 (24.6)	1.28 (0.72-2.27)
**Community size** ^**a**^									
1 500 000+	150 (25.1)	208 (35.7)	1.00 (reference)	48 (30.0)	61 (38.4)	1.00 (reference)	68(32.8)	71 (36.0)	1.00 (reference)
500 000–1 499 999	128 (21.4)	77 (13.2)	0.42(0.29-0.61)*	28 (17.5)	17 (10.7)	0.41(0.20-0.85)*	35 (16.9)	19 (9.55)	0.53(0.27-1.01)
100 000–499 999	151 (25.2)	146 (25.1)	0.68(0.50-0.93)*	39 (24.4)	37 (23.3)	0.68(0.37-1.24)	34 (16.4)	44(22.1)	1.22(0.70-2.15)
10 000 – 99 999	62 (10.4)	58 (9.97)	0.65(0.42-0.99)*	21 (13.1)	21 (13.2)	0.77(0.37-1.59)	29 (14.0)	32 (16.1)	1.08(0.59-1.97)
<10 000	108 (18.0)	93 (16.0)	0.60(0.42-0.86)*	24 (15.0)	23 (14.5)	0.71(0.35-1.44)	41 (19.8)	33 (16.6)	0.78(0.45-1.40)
**Biopsy type** ^**b**^									
FNA/Core	381(63.6)	237(40.7)	1.00 (reference)	110(68.8)	70(44.0)	1.00 (reference)	167(80.7)	92(46.2)	1.00 (reference)
Open	218(36.4)	345(59.3)	2.76(2.14-3.56)*	50 (31.3)	89 (56.0)	2.56(1.50-4.35)*	41 (19.3)	107 (53.8)	5.56(3.33-9.30)**
**Tumour size** ^**c**^									
<1.0	160 (27.4)	224 (39.2)	1.00 (reference)	30 (20.3)	54 (34.4)	1.00 (reference)	14 (7.54)	25 (13.7)	1.00 (reference)
1.0-1.5	125 (21.4)	143 (25.0)	0.80(0.58-1.11)	28 (18.92)	33 (21.0)	0.73(0.35-1.13)	17 (9.14)	29 (15.9)	0.96(0.36-2.54)
1.5-2.0	121 (20.7)	88 (15.4)	0.56(0.39-0.80)*	30 (20.3)	20 (12.7)	0.38(0.17-0.83)	27 (14.5)	35 (19.1)	0.84(0.33-2.12)
>2.0	179 (30.6)	117 (20.5)	0.50(0.36-0.69)**	60 (40.5)	50 (31.9)	0.56(0.30-1.08)	128 (68.8)	94 (51.4)	0.49(0.22-1.05)
**Stage at diagnosis** ^**c**^									
I	309 (53.5)	353 (64.2)	1.00 (reference)	65 (43.3)	85 (55.6)	1.00 (reference)	37 (19.1)	55 (28.4)	1.00 (reference)
II	194 (33.6)	138 (25.1)	0.66(0.50-0.87)*	51 (34.0)	39 (25.5)	0.80(0.45-1.43)	81 (41.8)	78 (41.9)	0.66(0.37-1.18)
III-IV	75 (13.0)	59 (10.7)	0.72(0.49-1.06)	34 (227)	29(19.0)	0.72(0.37-1.36)	76 (39.2)	53 (28.5)	0.46(0.25-0.85)*
**Nodal status** ^**c**^									
Negative	386 (67.6)	393 (72.6)	1.00 (reference)	93 (64.6)	98(67.1)	1.00 (reference)	83 (49.7)	99 (55.3)	1.00 (reference)
Positive	185 (32.4)	148 (27.4)	0.83 (0.63-1.08)	51 (35.4)	47 (32.4)	1.02(0.60-1.73)	84 (50.8)	80 (44.7)	0.79(0.48-1.27)

^a^models adjusted by: age at diagnosis (continuous) year of diagnosis (continuous).

^b^models adjusted by: age at diagnosis (continuous); year diagnosis (continuous); treatment center (categorical) tumour size (continuous); income quintile (categorical).

^c^models adjusted by: age at diagnosis (continuous); year diagnosis (continuous); treatment center (categorical); biopsy type at diagnosis (categorical); income quintile (categorical).

*p < 0.05.

**p < 0.0001.

Compared to women undergoing an FNA or core biopsy, women who underwent an open biopsy had significantly longer than median wait times to diagnosis irrespective of detection method; screen-detected (OR = 2.76, 95% CI = 2.14-3.56), interval (OR = 2.56, 95% CI = 1.50-4.35) and symptomatic (OR = 5.56, 95% CI = 3.33-9.30) (Table 2). Among screen-detected cancers, women diagnosed with larger (1.5-2.0 cm and >2.0 cm) compared to smaller tumours (<1.0 cm), had shorter diagnostic wait times (OR = 0.56 95% CI = 0.39-0.80 and OR = 0.50 95% CI = 0.36-0.69, respectively). Additionally, screen-detected breast cancers diagnosed with stage II and symptomatic cancers diagnosed at stage III or IV had significantly shorter diagnostic wait times compared to those diagnosed at stage 1 (OR = 0.66 95% CI = 0.50-0.87 and OR = 0.46, 95% CI = 0.25-0.85 respectively).

## Discussion

Overall, this study found that biopsy type was a major predictor of longer wait times to diagnosis, irrespective of detection method. Women having an open biopsy had a greater than median wait time to diagnosis, compared to women having a FNA or core biopsy. Larger tumour size and higher stage at diagnosis were associated with shorter diagnostic wait times for screen-detected cancers, while only higher stage was associated with shorter diagnostic intervals for symptomatic cancers. Interval cancers showed similar trends (although not significant) as screen-detected and symptomatic cancers.

We found that symptomatic detected breast cancers had a short diagnostic wait time. This result is expected as a large portion of these women present with breast symptoms which are suggestive of breast cancer, and may not have required imaging to confirm diagnosis, thus decreasing the interval to diagnosis. Our study also found that symptomatic cancers were diagnosed at higher stages than screen-detected cancers and more likely to have a biopsy as their first diagnostic procedure. Other studies similarly found women with abnormal mammograms had longer times to diagnosis (Brett et al. 1998;Burgess et al. 1998) or to treatment (Ramirez et al. 1999) than those presenting with breast abnormalities.

Although symptomatic breast cancers were more likely to be diagnosed among women living in lower compared to higher income areas, income quintile was not a predictor of diagnostic wait times for any of the detection groups. Numerous studies have failed to detect an association between delay to diagnosis and socio economic status (Burgess et al. 1998;Ramirez et al. 1999). Although studies out of the UK (Downing et al. 2007) and Ontario (Booth et al. 2010) found an association between low income quintile and increased risk of breast cancer related mortality, they did not examine diagnostic wait time as a predictor.

Among women with screen-detected breast cancers, those living in the smallest communities had shorter diagnostic wait times, compared to those living in the largest. This is consistent with a pan-Canadian study that found significantly shorter wait times to diagnosis in rural areas compared to urban for screen-detected cancers (Caplan et al. 2000). In explanation, it was postulated that women living in small towns that travel long distances for breast screening and receive abnormal results on a mammogram might be referred for a biopsy on the same day for convenience, while women living in larger towns with better access to care might be scheduled for a later time (Caplan et al. 2000). We also observed regional variation in diagnostic wait times for interval and symptomatic breast cancers. Compared to the South Central region, women who attended a treatment centre in the South Eastern region had shorter diagnostic wait times, while those in the Northern region were more likely to experience longer diagnostic wait times. Consistent with our results, a study conducted in Ontario found substantial regional variations in use of percutaneous biopsy over surgery as a first diagnostic procedure among women who were being investigated for a breast abnormality (Holloway et al. 2007).

We found that tissue diagnosis using open biopsy occurred frequently during the study period, despite the recommendation that tissue diagnosis of breast abnormalities be obtained prior to surgery (Bevers et al. 2009;McCready et al. 2005). Notably, symptomatic cancers were less likely to have an open diagnostic biopsy than screen-detected cancers. In contrast, a study conducted on women undergoing investigation for breast abnormalities in Ontario found previous mammography screening was associated with a lower use of open biopsy (Holloway et al. 2007). Unlike our study, their investigation included benign as well as malignant breast abnormalities, women outside the recommended ages for organized screening and did not distinguish between screen-detected and symptomatic cancers, all of which may explain the discrepancy in findings.

Women who had an open biopsy were substantially more likely to experience longer wait times to diagnosis than those having percutaneous FNA or core biopsy, irrespective of detection method. Another study in British Columbia found that women with screen-detected cancer who had open biopsies had double the diagnostic interval compared to those without biopsy (Olivotto et al. 2000). Interestingly, we found that women diagnosed with an interval cancer who received their last screen through OHIP (outside a screening program) were more likely to have longer wait times to diagnosis than those screened through the OBSP. This finding suggests a possibly different course of diagnostic work up for women undergoing screening and diagnosis through OHIP compared with the OBSP. The OBSP is associated with assessment centres that offer facilitated evaluation, including core needle biopsy of screen-detected abnormalities. Some women screened through OHIP and their providers may have reduced access to core needle biopsy, prompting greater use of open surgical biopsy for diagnosis. A study conducted in Ontario, showed a 20% improvement in achieving timely resolution of a mammogram detected abnormality requiring a core biopsy among women receiving their diagnostic work up through breast assessment affiliates compared to those receiving work up through family physicians (Quan et al. 2012). Shorter diagnostic intervals were also seen among women with screen-detected breast cancer in Manitoba who received diagnostic work up through direct referral by the Manitoba Breast Screening Program, compared to through usual care, although this study did not examine impact of biopsy type on the diagnostic interval (Decker et al. 2004). Organized approaches to screening, employed by programs like the OBSP, offer advantages such as coordinated follow-up of women who have abnormal screening results and as well as standardized approaches to diagnostic work-up (Quan et al. 2012).

Women diagnosed with tumours at a larger size and greater stage had shorter wait times to diagnosis, compared to those diagnosed at earlier stages, and with smaller tumours. This finding is consistent with literature on the expedition process of more suspect cases, which showed tumours labeled as more suspicious were associated with decreased likelihood of diagnostic delay independent of other factors (Olivotto et al. 2002;Ganry et al. 2004;Caplan et al. 2000). Other research has shown women diagnosed with invasive cancer experienced shorter intervals than those eventually diagnosed with a benign lesion or ductal carcinoma in-situ (Olivotto et al. 2001;Chiarelli et al. 2005).

Strengths of this study include the large number of breast cancer cases allowing us to stratify our analysis by detection method. As well, information on breast cancer diagnosis was obtained through the OCR. Evaluation of the OCR suggests a high level of completeness and accuracy for breast cancer ascertainment (Holowaty et al. 1995). Furthermore, through chart abstraction, we were able to obtain detailed information on type and dates of assessment procedure and prognostic tumour characteristics of the diagnosed breast cancer cases.

Several limitations should be addressed. It is possible that access to core needle biopsy for screen-detected cancers has increased since the time period of this study, thereby reducing the time to diagnosis in this group. Although we would be interested in examining the impact of longer diagnostic intervals on breast cancer survival, we do not have survival information for the present study. This study could not distinguish between patient level and system level factors associated with delays along the diagnostic pathways. Although the present study examined wait times to diagnosis of breast cancer, the interval from diagnosis to treatment is also an important factor which may contribute to overall survival of women with breast cancer. A future proposed study will examine factors associated with treatment intervals by detection method.

Evaluating factors associated with wait times to breast cancer diagnosis provides targets for intervention that may ultimately improve the prognosis for women. Our study identified both clinical and prognostic factors associated with diagnostic wait times among women who presented with screen-detected, interval and symptomatic breast cancers. Most notably, open surgical biopsy was a common method of tissue diagnosis for breast cancer in Ontario during the study period and was a major predictor of having a greater than median wait time to diagnosis, irrespective of detection method. Our study was consistent with others that reported expedited diagnostic work-up for breast cancers with more advanced prognostic features. Interventions to facilitate alignment of diagnostic approaches with best practices will improve the process of providing a breast cancer diagnosis.
